# Activation of NLRP3 Inflammasome in Liver of Long Evans Lactating Rats and Its Perinatal Effects in the Offspring after Bisphenol F Exposure

**DOI:** 10.3390/ijms241814129

**Published:** 2023-09-15

**Authors:** Beatriz Linillos-Pradillo, Sergio D. Paredes, María Ortiz-Cabello, Margret Schlumpf, Walter Lichtensteiger, Elena Vara, Jesús A. F. Tresguerres, Lisa Rancan

**Affiliations:** 1 Department of Biochemistry and Molecular Biology, School of Medicine, Complutense University of Madrid, 28040 Madrid, Spain; beatlini@ucm.es (B.L.-P.); mariaoc00@gmail.com (M.O.-C.); evaraami@ucm.es (E.V.); 2 Department of Physiology, School of Medicine, Complutense University of Madrid, 28040 Madrid, Spain; spared01@ucm.es (S.D.P.); guerres@ucm.es (J.A.F.T.); 3 GREEN Tox and Institute of Veterinary Pharmacology and Toxicology, University of Zurich, CH-8057 Zurich, Switzerland; margret.schlumpf@access.uzh.ch (M.S.); walter.lichtensteiger@access.uzh.ch (W.L.)

**Keywords:** Bisphenol F, RNS, NLRP3 inflammasome, liver, offspring

## Abstract

The liver is the organ responsible for the metabolism and detoxification of BPF, the BPA analogue that is replacing it in plastic-based products. It is not known whether BPF can trigger inflammatory responses via the NLRP3 inflammasome, which plays a major role in the development of liver disease. The aim of this study was to evaluate nitrosative stress species (RNS) and NLRP3 inflammasome activation in the liver of lactating dams after BPF exposure. Moreover, it was studied whether this effect could also be observed in the liver of female and male offspring at postnatal day 6 (PND6). 36 Long Evans rats were randomly distributed according to oral treatment into three groups: Control, BPF-low dose (LBPF; 0.0365 mg/kg b.w./day) group and BPF-high dose (HBPF; 3.65 mg/kg b.w./day) group. The levels of nitrosative stress-inducing proteins (eNOS, iNOS, HO-1d), NLRP3 inflammasome components (NLRP3, PyCARD, CASP1) and proinflammatory cytokines (IL-1β, IL-18, IFN-γ and TNF-α) were measured by gene and protein expression in the liver of lactating dams and in female and male PND6 offspring. Lactating dams treated with LBPF showed a significant increase in iNOS and HO-1d, activation of NLRP3 components (NLRP3, PyCARD, CASP1) and promoted the release of proinflammatory cytokines such as IL-1β, IL-18, IFN-γ and TNF-α. Similar effects were found in female and male PND6 offspring after perinatal exposure. LBPF oral administration and perinatal exposure caused an increase of nitrosative stress markers and proinflammatory cytokines. Also, NLRP3 inflammasome activation was significantly increased in in the liver of lactating dams and PND6 offspring.

## 1. Introduction

Nowadays it is well documented that bisphenol A (BPA) exposure can cause liver tissue remodeling and fibrosis due to the generation of reactive oxygen species (ROS) and an uncontrolled inflammatory cascade [1]. This liver injury can lead to diseases such as hepatic steatosis, tumors, and metabolic syndrome. An important role of the NLRP3 inflammasome has been described in liver diseases [2,3]. Inflammasomes are key components of the natural immune system that can largely protect normal liver functions against pathogenic infections, metabolic diseases, and cellular stress [4]. NLRP3 inflammasome is a multiprotein scaffold that responds to damage-associated molecular patterns (DAMPs) and can mediate the catalytic activation of caspase-1 (CASP1), promoting the cleavage and release of IL-1β and IL-18 [5]. However, excessive inflammatory response regulated by NLRP3 inflammasome triggers liver disease progression [4].

Previous studies showed that BPA promoted inflammation and fibrosis progression with a key role of the NLRP3 inflammasome in the liver of obese mice after BPA and high-fat diet administration [6]. Knockout mouse models suggested that inhibition of the NLRP3 inflammasome reduced liver inflammation, indicating that the NLRP3 inflammasome is involved in the progression of non-alcoholic fatty liver disease (NAFLD) [7,8]. Furthermore, NLRP3 upregulation and increased gene and protein expression of IL-1β, IL-18, NLRP3, and CASP1 were observed in laying hens after high doses of BPA [9].

Due to the large number of studies demonstrating the health risks of BPA, the development and production of alternatives to this endocrine-disrupting chemical (EDC), has been stimulated to replace it in a myriad of applications [10]. Some of the new alternatives to BPA are the bisphenol analogues, such as bisphenol F (BPF). BPF is a diphenylalkane with two phenol rings linked through a methylene. BPF is replacing BPA in the manufacture of plastic-based products [11]. Also, BPF is the predominant bisphenol found in foodstuffs, representing 17% of total bisphenols.

After oral absorption, BPF is mainly metabolized in the liver by BPF-glucuronide and BPF-sulfate. Most BPF is excreted in the urine as a sulfate conjugate. Nonetheless, between 7–9% remains in the rat tissues 96 h after BPF exposure [12]. The liver seems to be more vulnerable to the effect of lower doses of bisphenols as it is responsible for the metabolism and detoxification of compounds to maintain homeostasis in the whole organism. It also plays an indispensable role in mediating inflammatory responses [13]. It is particularly interesting to investigate and understand how exposure to different EDCs can affect the developmental period. This is because an unborn fetus, as well as the placenta, is vulnerable because of the lack of the proper enzymatic machinery. This makes the gestation and the perinatal period, the most vulnerable times to EDC toxicity in human life [14]. In addition, effects may manifest differently in males and females due to differences in metabolism, storage, and elimination of xenobiotics [15].

Previous studies by our research group showed that low-dose BPF increased oxidative stress by reducing antioxidant enzyme activities and altering the glutathione system in lactating rats and their offspring [16]. However, it is unknown whether BPF triggers NLRP3 inflammasome-mediated inflammatory responses in the liver.

The aim of this study was to evaluate nitrosative stress after BPF exposure, and whether reactive nitrogen species (RNS) could serve as a stimulus for NLRP3 inflammasome activation and generation of inflammation and apoptosis in the liver of lactating dams. Moreover, it was studied whether this effect could also be observed in the liver of female and male offspring at postnatal day 6 (PND6).

## 2. Results

In addition to reactive oxygen species (ROS), there are also reactive nitrogen species (RNS) that are produced physiologically. However, imbalances between the production and neutralization of these RNS are known as nitrosative stress.

**Figure 1:**
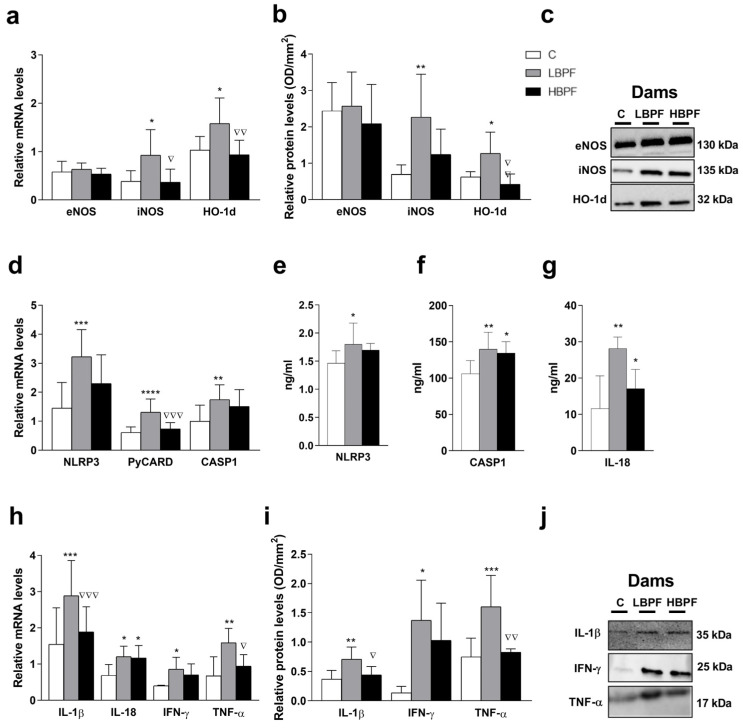
BPF effect on nitrosative stress markers, NLRP3 inflammasome activation and release of pro-inflammatory cytokines in the liver of lactating dams. (**a**) mRNA levels of eNOS, iNOS and HO-1d; (**b**) protein expression of eNOS, iNOS and HO-1d; (**c**) representative eNOS, iNOS and HO-1d protein blots measured by Western blotting; (**d**) mRNA levels of NLRP3 inflammasome components (NLRP3, PyCARD and CASP1); (**e**) NLRP3, (**f**) CASP1, and (**g**) IL-18 protein levels measured by ELISA; (**h**) mRNA levels of pro-inflammatory cytokines IL-1β, IL-18, IFN-γ and TNF-α; (**i**) protein expression of IL-1β, IFN-γ and TNF-α; and (**j**) representative IL-1β, IFN-γ and TNF-α protein blots evaluated by Western blotting. Data represent mean ± SD. *n* = 6 lactating control (C) dams; *n* = 6 lactating BPF low-dose (LBPF)-treated dams; *n* = 10 lactating BPF high-dose (HBPF)-treated dams. For qRT-PCR analysis, three replicates for each sample were performed. For protein, *n* = 5 rats per experimental group. Statistical significance was determined by one-way ANOVA. * *p* < 0.05; ** *p* < 0.01; *** *p* < 0.001; **** *p* < 0.0001 compared to control group. ∇ *p* < 0.05; ∇∇ *p* < 0.01; ∇∇∇ *p*  <  0.001, LBPF vs. HBPF.

When lactating dams were treated with LBPF, gene and protein levels of oxidative stress-inducing proteins such as iNOS and HO-1d were significantly increased compared to the control group. In addition, iNOS and HO-1d mRNA and protein levels of HO-1d were higher in the LBPF group as compared with the HBPF-treated dams. No significant changes were shown in the physiological eNOS isoform after the administration of both doses of BPF in the liver of lactating dams (Figure 1a,b). To further investigate the role of BPF on hepatic inflammation, we measured the mRNA and protein levels of NLRP3 inflammasome components. The mRNA of NLRP3, PyCARD (ASC adaptor), and CASP1 were upregulated in LBPF-treated dams when compared to the control group. Higher PyCARD mRNA levels were also shown after LBPF administration as compared to HBPF in the liver of lactating dams (Figure 1d). Higher protein expression of NLRP3, CASP1, and IL-18 were obtained after LBPF administration when compared to control dams (Figure 1e–g). CASP1 and IL-18 protein expression levels were also higher in HBPF when compared to control dams (Figure 1f,g). Regarding proinflammatory cytokines IL-1β, IL-18, IFN-γ, and TNF-α, they were considerably upregulated in LBPF-treated dams, whereas no significant change was observed in the HBPF group as compared with the control group except for IL-18 mRNA levels. Significant differences were also observed between both treatment groups, resulting in higher gene and protein levels of IL-1β and TNF-α in the LBPF group (Figure 1h,i). Representative protein blots for each tested marker are shown in Figure 1c,j.

Hence, LBPF increased nitrosative stress levels, which could be the stimuli to activate the NLRP3 inflammasome and to promote inflammatory responses in the liver of lactating dams. 

**Figure 2:**
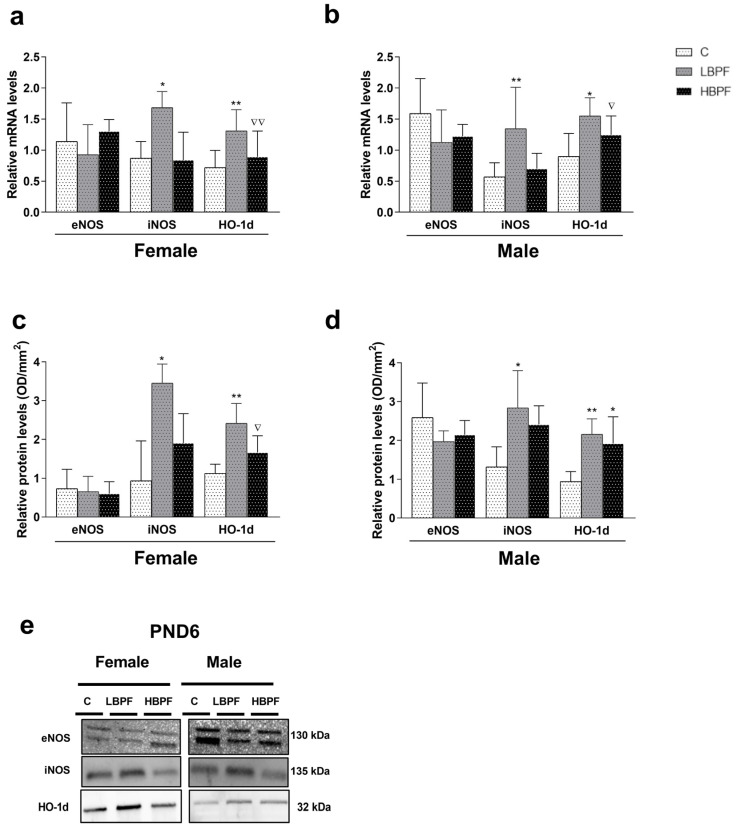
BPF pre- and perinatal effect on nitrosative stress markers in the liver of female and male PND6 offspring. (**a**) mRNA levels of eNOS, iNOS and HO-1d in female offspring; (**b**) mRNA levels of eNOS, iNOS and HO-1d in male offspring; (**c**) protein expression of eNOS, iNOS and HO-1d in female offspring; (**d**) protein expression of eNOS, iNOS and HO-1d in male offspring; and (**e**) representative eNOS, iNOS and HO-1d protein blots measured by Western blotting in both sexes. Data represent mean ± SD. For mRNA analysis, *n* = 12 female PND6 pups and *n* = 12 male PND6 pups for each experimental group with three replicates for each sample, control (C), low-dose BPF (LBPF) and high-dose BPF (HBPF), were evaluated, and for protein analysis, *n* = 5 female and *n* = 5 male per experimental group. Statistical significance was determined by one-way ANOVA. * *p*  <  0.05; ** *p*  <  0.01 compared to control group. ∇ *p* <  0.05; ∇∇  <  0.01, LBPF vs. HBPF.

To study whether perinatal administration of BPF generated alteration of the nitrosative balance in the liver of female and male offspring, we evaluated the same isoforms of NO and HO-1d. When female PND6 offspring was pre- and perinatally exposed to LBPF, the mRNA and protein levels of iNOS and HO-1d were increased in the LBPF group as compared to the control group (Figure 2a,c). Also, higher levels of HO-1d mRNA and protein expression were observed in LBPF-exposed female offspring compared to the HBPF group (Figure 2a). Notably, eNOS isoform showed no differences between groups (Figure 2a,c).

In males exposed pre- and perinatally to BPF, the same results were obtained as in females. Thus, iNOS and HO-1d gene and protein levels increased in the LBPF-treated males compared to the control group, and no significant changes in eNOS isoform between groups were found (Figure 2b,d). Also, higher levels of HO-1d mRNA were observed in LBPF-exposed male offspring as compared to HBPF (Figure 2b). HO-1d protein levels were higher in HBPF compared to the control group. Figure 2e shows eNOS, iNOS, and HO-1d representative blots analyzed in both PND6 females and males. In both sexes, there was also an enhanced expression of the inducible HO-1d and iNOS isoforms, which increased nitrosative stress levels (Figure 2).

**Figure 3:**
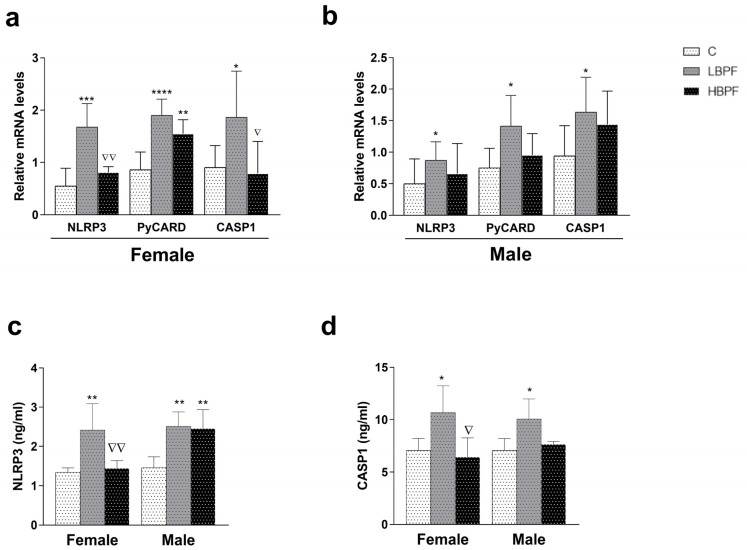
BPF pre- and perinatal effect on NLRP3 inflammasome induction in the liver of female and male PND6 offspring. (**a**) mRNA levels of NLRP3, PyCARD and CASP1 in female offspring; (**b**) mRNA levels of NLRP3, PyCARD and CASP1 in male offspring; (**c**) NLRP3, (**d**) CASP1 protein levels in male and female offspring measured by ELISA. Data represent mean ± SD. For mRNA analysis, *n* = 12 female PND6 pups and *n* = 12 male PND6 pups for each experimental group with three replicates for each sample, control (C), low-dose BPF (LBPF) and high-dose BPF (HBPF), were evaluated, and for protein analysis, *n* = 5 female and *n* = 5 male per experimental group. Statistical significance was determined by one-way ANOVA. * *p*  <  0.05; ** *p*  <  0.01; *** *p*  <  0.001; **** *p*  <  0.0001 compared to control group. ∇ *p*  <  0.05; ∇∇ *p*  <  0.01; LBPF vs. HBPF.

Regarding the NLRP3 inflammasome pathway activation, an increase in NLRP3 gene expression and the following up-regulation of the adaptor ASC (PyCARD) and CASP1 mRNAs were shown after LBPF administration in female offspring (Figure 3a). In addition, increased PyCARD mRNA levels were observed in HBPF-exposed female offspring when compared to the control group (Figure 3a). Higher levels of NLRP3 and CASP1 mRNA and protein expression were observed in LBPF-exposed female offspring compared to the HBPF group (Figure 3a,c,d).

When PND6 male offspring was pre- and perinatally exposed to LBPF, an increase in NLRP3, PyCARD, and CASP1 was observed as compared to the control group (Figure 3b). This was also observed with respect to the protein expression of NLRP3 and CASP1 (Figure 3c,d). NLRP3 protein expression was also upregulated in HBPF-treated offspring when compared to control male offspring (Figure 3c). Notably, NLRP3 pathway activation occurred in both sexes, allowing binding to the adaptor molecule and promoting CASP1 gene expression after pre- and perinatal exposure to LBPF (Figure 3).

**Figure 4:**
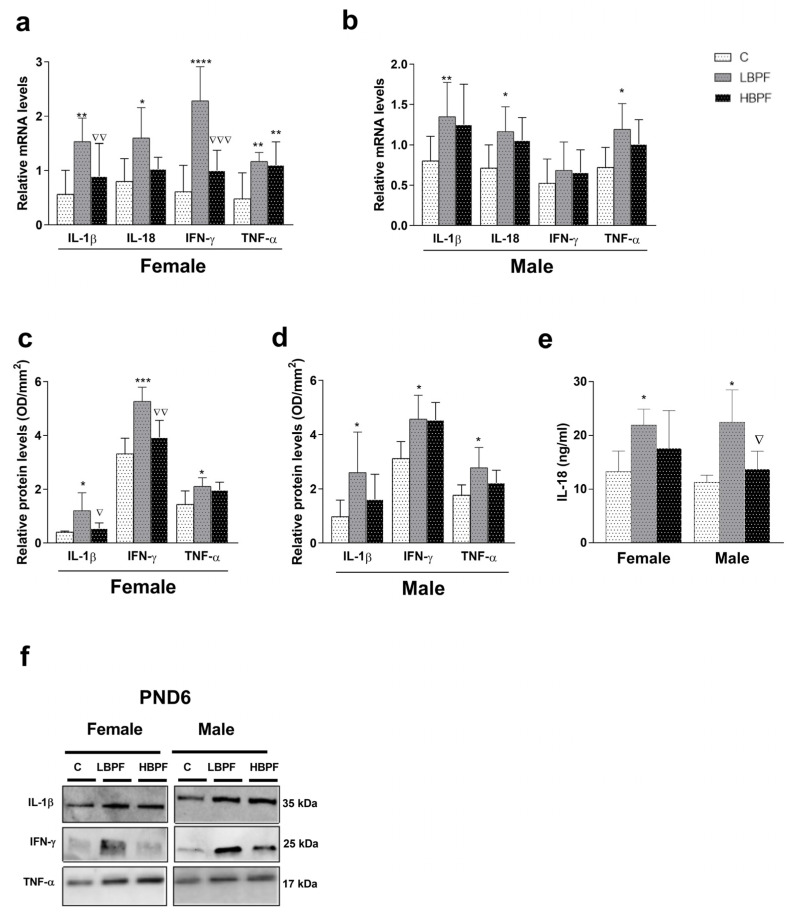
BPF pre- and perinatal effect on release of pro-inflammatory cytokines in the liver of female and male PND6 offspring. (**a**) mRNA levels of IL-1β, IL-18, IFN-γ and TNF-α in female offspring; (**b**) mRNA levels of IL-1β, IL-18, IFN-γ and TNF-α in male offspring; (**c**) protein expression of IL-1β, IFN-γ and TNF-α in female offspring; (**d**) protein expression of IL-1β, IFN-γ and TNF-α in male offspring; (**e**) IL-18 protein levels in male and female offspring measured by ELISA and (**f**) representative IL-1β, IFN-γ and TNF-α protein blots measured by Western blotting in both sexes. Data represent mean ± SD. For mRNA analysis, *n* = 12 female PND6 pups and *n* = 12 male PND6 pups for each experimental group with three replicates for each sample, control (C), low-dose BPF (LBPF) and high-dose BPF (HBPF), were evaluated, and for protein analysis, *n* = 5 female and *n* = 5 male per experimental group. Statistical significance was determined by one-way ANOVA. * *p*  <  0.05; ** *p*  <  0.01; *** *p*  <  0.001; **** *p*  <  0.0001 compared to control group. ∇ *p*  <  0.05; ∇∇ *p*  <  0.01; ∇∇∇ *p*  <  0.001, LBPF vs. HBPF.

When female PND6 offspring was pre- and perinatally exposed to LBPF, the mRNA and protein levels of IL-1β, IL-18, IFN-γ, and TNF-α were increased when compared to the control group (Figure 4a,c,e). Also, higher mRNA and protein levels of IL-1β and IFN-γ were observed in LBPF-exposed female offspring when compared to the HBPF group (Figure 4a,c). TNF-α mRNA levels were upregulated in HBPF-exposed female offspring compared to the control group (Figure 4a).

In males exposed pre- and perinatally to BPF, up-regulated mRNA levels of IL-1β, IL-18 and TNF-α were observed as compared to the control group (Figure 4b). Protein levels of IFN-γ, IL-1β, and TNF-α were also higher in LBPF-exposed male offspring as compared with the control group (Figure 4d). IL-18 protein levels were also higher in LBPF-exposed animals when compared to control and HBPF-exposed male offspring (Figure 4e). Figure 4f shows representative blots of pro-inflammatory cytokines in PND6 females and males.

**Figure 5:**
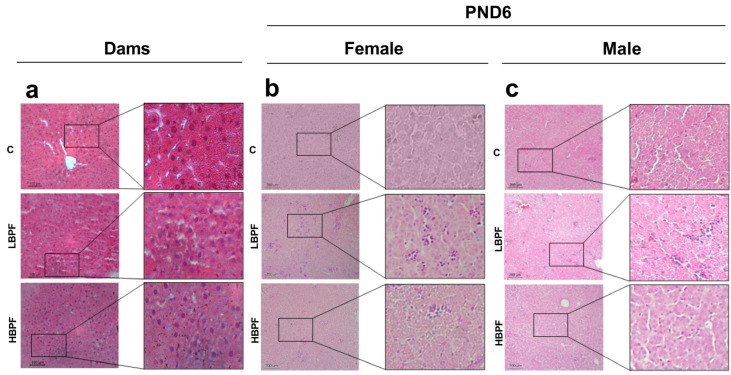
Histological study after BPF exposure of liver from (**a**) dams, (**b**) female and (**c**) male PND6 offspring stained with H&E. Representative images from control, LBPF and HBPF liver (10×) and magnified image of the specific tissue section (20×) indicating the aggregation of nuclei.

Regarding the histological study of the liver of lactating dams, no changes were still observed in cellular structure in the livers of BPF-treated dams compared to control hepatocyte images (Figure 5a). However, in both sexes of offspring, BPF administration induced nuclei aggregation and inflammatory cell infiltration in the liver of PND6 offspring compared to control pups with more noticeable effects at LBPF (Figure 5b,c).

After BPF exposure, NLRP3 inflammasome activation and pro-inflammatory cytokines release were observed in offspring of both sexes. These same effects were observed in the liver of lactating dams with more noticeable effects after LBPF exposure.

## 3. Discussion

Oxidative stress and inflammation in the liver are closely correlated, as they occur simultaneously and interact with each other and are crucial in the initiation and development of liver disease [13].

In a previous study by our research group, antioxidant enzyme activities were decreased, and oxidized glutathione levels were increased after low doses of BPF in lactating Long Evans rats and their offspring, in addition to increased lipid peroxidation. Thus, LBPF increases oxidative stress [16]. However, it was unknown whether BPF could increase nitrosative stress and serve as a stimulus to trigger inflammatory responses after administration of two doses of BPF: a low dose of 0.0365 mg/kg/b.w./day (LBPF) and a high dose of 3.6 mg/kg/b.w./day (HBPF) in the liver of lactating dams and PND6 offspring after pre- and perinatal BPF exposure.

Among the reactive nitrogen species (RNS), nitric oxide (NO) is a signaling molecule involved in many biological processes: blood pressure, inhibition of platelet aggregation, and neurotransmission; synthesized by at least three isoforms: neuronal nNOS, endothelial eNOS, and inducible iNOS. NO overproduction is associated with enhanced RNS production, which is able to induce structural damage to biomolecules, including proteins, lipids, and DNA [17].

No significant changes were found in the constitutive eNOS isoform, but increased gene and protein expression of inducible iNOS in LBPF-treated dams was observed. Excess of NO levels from increased iNOS activity can cause liver cell injury due to nitrosylation of thiol residues of many cellular enzymes, as well as a triggering effect of innate and adaptive immune responses [18]. Increased gene and protein expression of inducible HO-1d were also observed in LBPF-treated dams. HO-1d responds to transcriptional induction due to alterations in oxygen tension, inflammatory mediators, heat shock, oxidative stress, and NO levels. Therefore, HO-1d induction is elevated after nitrosative stress in order to prevent further injury [19].

Increased mitochondrial reactive oxygen species (ROS) and RNS are able to influence several physiological and pathological processes, including inflammation. Inflammation may be triggered by several different processes being the activation of the inflammasome one of the most important. The NLRP3 inflammasome can be activated in response to a wide range of stimuli such as infection, tissue damage, or metabolic stress (via different pathways: ATP, damaged mitochondria, lysosomal breakdown, changes in Ca^2+,^ K^+^, and also increases in mitochondrial and non-mitochondrial ROS concentrations). Once NLRP3 is activated, it binds to the adaptor molecule PyCARD (ASC; apoptosis-associated speck-like protein containing a CARD), which recruits and activates procaspase-1 into caspase-1 (CASP1), which is able to promote the maturation of proinflammatory cytokines such as IL-1β and IL-18. In addition, CASP1 is able to cleave protein precursors that affect the cell cytoskeleton, glycolysis, mitochondrial function, and inflammation [20]. It also induces pyroptosis, an inflammatory form of programmed cell death [21].

An increase in gene expression of the NLRP3 sensor, its adaptor molecule PyCARD, and CASP1, the three components of the NLRP3 inflammasome were observed in LBPF-treated dams. In turn, a release of proinflammatory cytokines such as IL-1β, IL-18, IFN-γ, and TNF-α occurred after exposure to LBPF, as measured by gene and protein expression in the liver of lactating dams.

IL-1β and IL-18, members of the IL-1 superfamily of cytokines, promote processes associated with infection, inflammation, and autoimmunity. IL-1β is key in the activation of hepatic stellate cells (HSC) and promotes the recruitment of inflammatory cells, contributing to fibrosis and triglyceride accumulation in hepatocytes and their death together with TNF-α [3]. TNF-α causes hepatic inflammation, proliferation, and apoptosis, as well as changes in HSC morphology [22]. TNF-α can also promote the recruitment of proinflammatory neutrophils and macrophages and the activation of fibrogenic pathways leading to the development of liver fibrosis [23].

IL-18 induces IFN-γ synthesis, in addition to activating NK cells and cytotoxic T lymphocytes, and seems to be involved in modulating the gut microbiota [3]. IFN-γ is a regulatory mechanism of the NLRP3 inflammasome and has a dual role: it activates effector cells such as NK lymphocytes and also tends to decrease activation through iNOS because NO induces nitrosylation of the NLRP3 protein and can inhibit its activity after a prolonged time [24]. The results obtained in the liver of lactating dams are consistent with a study that showed a significant increase in the levels of TNF-α and other inflammatory molecules in zebrafish after administration of BPF between 10–1000 µg/L [25].

Therefore, oral administration of LBPF to lactating dams led to an increase in liver RNS, which could stimulate the NLRP3 inflammasome and promote the release of pro-inflammatory cytokines.

There are no previous studies showing the influence of BPF on the activation or inhibition of inflammasomes, their components, or the release of products, but there is already data about the effects of BPA administration, as previously mentioned [6,9]. In a recent study by our research group, it was shown that after administration of low doses of BPA, oxidative stress and NO levels increased, with a decrease in the endogenous antioxidant enzyme system (CAT, SOD, GST, GR, and GST) and glutathione system (GSSG/GSH ratio) in lactating dams as well as in female offspring [26]. Therefore, understanding how BPF exposure can affect the developmental period is very important, as it is the most critical and vulnerable period in human life. This exposure could cause a higher risk of developing diseases in adulthood due to their limited ability in this period of life to metabolize and process these chemicals [14,27]. Also, it is the moment in which the brain, as well as other organs, are in the phase of development.

Furthermore, human placental cells incubated with BPA and BPF are shown to activate the P2X7 receptor, promoting the NLRP3 inflammasome and increasing the activity of several caspases, showing a toxic effect. This could trigger preterm birth and pre-eclampsia in humans [28]. BPF administration also increases spontaneous abortions in pregnant dams in a dose-dependent manner [29].

Our results in PND6 offspring showed an increase in gene and protein expression of iNOS and no change in the eNOS isoform in both males and females, as well as an increase in inducible mRNA and protein HO-1d levels in both sexes. In a previous study [16], higher levels of the GSSG/GSH ratio were found in females than in males, but antioxidant enzymes were decreased in both sexes.

Regarding the components of the inflammasome, in both female and male offspring, an increase in NLRP3, PyCARD, and CASP1 was observed after pre- and perinatal exposure to LBPF, together with the consequent release of proinflammatory cytokines IL-1β, IL-18, IFN-γ and TNF-α. Therefore, one of the stimuli responsible for the activation of NLRP3 components and the release of inflammation-promoting cytokines may be the excess of RNS after exposure to this chemical.

In addition, inflammatory cell infiltration and aggregation was observed more noticeable after LBPF in both female and male offspring. However, no notable morphological changes were observed in lactating dams during exposure. Liver damage following perinatal exposure to LBPF was also observed in other studies [30,31]. This may be due to the fact that after perinatal exposure, the fetus is in the process of tissue ontogeny, being much more vulnerable to such chemical exposure, and on postnatal day 6 (PND6), structural alterations are already observed with aggregation of nuclei and infiltration of inflammatory cells in the liver. Therefore, this makes the fetus much more sensitive and vulnerable to the effect of BPF on the liver than adult dams.

Finally, the administration of LBPF had more noticeable effects than HBPF in the liver of lactating dams and their offspring. This might be due to the particular behavior of bisphenol in dose-response curves, so it may also be interesting to evaluate and analyze the effects of BPF, as well as other BPA analogues, at very low concentrations, typical of environmental exposure [32] on other organs apart from the liver. However, further research on the effect of BPF on inflammation and its mechanisms of inflammasome activation would be needed.

## 4. Materials and Methods

### 4.1. Animals and Treatments

After 10 days of acclimatization, 36 female (8 weeks of age) and 18 male (10 weeks of age) Long Evans rats (Janvier Labs, Le Genest-Saint-Isle, France) were randomly divided into three groups: control group (non-treated), low dose (0.0365 mg/kg body weight/day; LBPF) group of BPF and high dose (3.65 mg/kg body weight/day; HBPF) group of BPF. In each experimental group, there were 12 females and 6 males. Except for the control group, which received chow with a corresponding concentration of corn oil, all groups were fed their corresponding diet with BPF, and the experiment lasted 60 days. Food and water were fed ad libitum. The doses of BPF used were chosen according to previous studies on BPA [26,33] and the large existing literature, where the dose range of BPA (2.5–50 mg/kg) induced impairment learning and memory loss in rodents when BPA was administered in the perinatal period. Thus, the high dose is 3.65 mg/kg higher than 2.5 mg/kg; while the low dose was 100 times lower, to investigate whether, even with such a small dose, any effects were observed.

All experimental procedures in this study were in accordance with the Guidelines for Ethical Care of Experimental Animals of the European Union (2010/63/UE) approved by the Ethical Committee of the Complutense University of Madrid (Madrid, Spain). This research is within a European project entitled “Novel Testing Strategies for Endocrine Disruptors in the Context of Developmental NeuroToxicity”, supported by the European Union’s Horizon 2020 Research and Innovation Programme (ENDpoiNTs project; grant number: 825759).

### 4.2. Chemicals and Experimental Design

BPF with purity > 99% was purchased from Sigma Aldrich (Buchs, Switzerland) (CAS Number 620-92-8; article number: 239658). It was dissolved in ethanol and then in corn oil at a ratio of 10% ethanol and 90% corn oil. The chosen rat chow was purchased from Granovit AG (Kaiseraugst, Switzerland) and corresponds to a diet with natural ingredients low in phytoestrogens.

Rats were housed in special polypropylene cages (Sodispan Research, Coslada, Madrid, Spain), water bottles were made of glass, and a cylindrical environmental enrichment element was included. In vivo experimental design consisted of five phases: premating (2 weeks), mating (10 days), pregnancy (23 days), lactation (6 days) and dissections. During premating, female and male rats were treated with a control diet or the corresponding dose of BPF in the diet for 2 weeks. After checking that the female was in the estrus phase, the mating phase took place between a male and a female from the same group. The following morning, a check for a sperm-positive vaginal smear or sperm-plug was carried out and the process was repeated all mornings for 10 days. Diet treatment was maintained during the whole pregnancy period. Six females were pregnant in the control and LBPF groups, and 10 females were pregnant in the HBPF group. Before the birth of the offspring, pregnant dams were separated into individual cages for lactation, and dietary treatment was maintained until postnatal day 6 (PND6). During all phases of the in vivo experiment, the cages of the control group were kept separate from the BPF-treated groups to avoid any possibility of spreading chow containing BPF.

**Figure 6:**
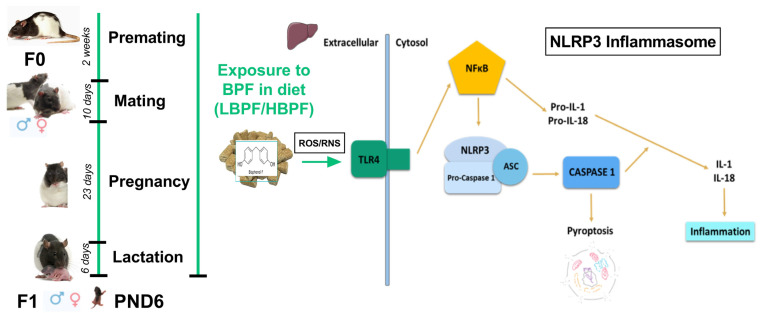
Experimental design. Parental generation (F0) was exposed to a diet containing a low dose (LBPF; 0.0365 mg/kg body weight/day) or a high dose (HBPF; 3.65 mg/kg body weight/day) of BPF or received a control diet (C) during the entire experiment. The levels of nitrosative stress and the NLRP3 inflammasome pathway in the liver of lactating dams and their offspring after BPF administration were studied. Activation of the NLRP3 inflammasome ultimately resulted in the release of the interleukins IL-1β, IL-18, IFN-γ and TNF-α, and could be triggered by different stimuli, including the generation of reactive oxygen species and nitrogen species (ROS/RNS). Figure created with Prism v7 (GraphPad Software Inc., San Diego, CA, USA).

Lactating dams were sacrificed by decapitation using a guillotine. Female and male offspring were sacrificed at PND6 by decapitation using scissors. The livers were collected and immediately frozen in liquid nitrogen and stored at −80 °C until analysis (Figure 6).

### 4.3. RNA Isolation and Quantitative Real-Time PCR (qRT-PCR) Analysis

Total RNA was isolated from liver tissues by using TRI Reagent Kit (Molecular Research Center, Inc., Cincinnati, OH, USA) and reverse transcribed into cDNA by using the StaRT Reverse Transcription Kit (AnyGenes, Paris, France). qRT-PCR was performed using a 7500 Fast Real Time PCR System thermal cycler (Applied Biosystems, MA, USA) according to the instruction of the TB Green^®^ Premix Ex Taq™ (Takara Bio Inc., Shiga, Japan). The related mRNA expression was normalized to 18s mRNA, and qRT-PCR data were analyzed using the comparative 2^-△△Ct^ method [34]. The following primers were used to amplify rat genes: forward (F) primer 5′-CCAGTGCCCTGCTTCATC-3′ and reverse (R) primer 5′GCAGGGCAAGTTAGGATCAG-3′ for eNOS, F primer 5′-CTTTGCCACGGACGAGAC-3′ and R primer 5′-TCATTGTACTCTGAGGGCTGAC-3′ for iNOS, F primer 5′-GTCAAGCACAGGGTGACAGA-3′ and R primer 5′-ATCACCTGCAGCTCCTCAAA-3′ for HO-1d, F primer 5′ TGAAAGCCTAGAAAGTCTGAAGAAC-3′ and R primer 5′-CGTGTTACCGTCCTTTTGC -3′ for IFN-γ, and F primer 5′- GGTGCATGGCCGTTCTTA-3′ and R primer 5′-TCGTTCGTTATCGGAATTAAC-3′ for 18S. The other rat primers (NLRP3, PYCARD, CASP1, IL-1β, IL-18, TNF-α) were custom primers and validated (AnyGenes, Paris, France).

### 4.4. Protein Preparation and Western Blot Analysis

Livers were homogenized with modified RIPA lysis buffer (PBS, Igepal, Sodium deoxycholate (D5670-5G), 10% SDS, PMSF, 0.5 M EDTA and 100 mM EGTA) to which protease inhibitor cocktail (Sigma #P-2714), PMSF (#P7626, 1 mM), sodium orthovanadate (#S6506, 2 mM) and sodium pyrophosphate (#S6422, 20 mM) were added. Samples were sonicated and boiled for 10 min at 100 °C in a ratio of 1:1 with gel-loading buffer (100 mmol/L Tris HCl [pH 6.8], 4% SDS, 20% glycerol, bromophenol blue 0.1, 200 mmol/L dithiothreitol). Total protein lysates (25 μg of dams’ sample and 50 μg of female and male offspring sample) were subjected to SDS-PAGE by using 10% Mini-PROTEAN^®^ TGX™ Precast acrylamide Gels (Bio-Rad Laboratories, Richmond, CA, USA). After electrophoresis, Stain Free technology was activated using the BioRad^®^ ChemiDoc MP Imaging System (Bio-Rad Laboratories, Richmond, CA, USA) and was transferred onto a PVDF membrane using Trans-Blot^®^ Turbo™ Transfer System (Bio-Rad Laboratories, Richmond, CA, USA).

The obtained membrane filter was then blocked with a blocking buffer containing 5% non-fat milk in 20 mM Tris pH 7.5, 150 mM NaCl, and 0.01% Tween-20 at 37 °C for 1 h. Rabbit polyclonal primary antibodies (dilution 1:1000) for immunoblotting were as follows: anti-eNOS (#PA1-037, Thermo Fisher Scientific, Waltham, MA USA), anti-IL-1β (#PA5-95455, Thermo Fisher Scientific, Waltham, MA, USA), anti-HO-1d (#3391, BioVision, Milpitas, CA, USA), anti-iNOS (#AB16311, Chemicon International, Temecula, CA, USA), anti-IFN-γ (#40499, Signalway Antibody, College Park, MD, USA) and anti-TNF-α (#500-P72, PeproTech EC, Ltd. London, UK) for 12 h at 4 °C, followed by incubation with a goat anti-rabbit IgG secondary antibody (dilution 1:7000; Santa Cruz Biotechnology, Inc., Santa Cruz, CA, USA).

Protein detection was performed using the Clarity Western ECL Substrate assay kit (Bio-Rad Laboratories, Richmond, CA, USA) by chemiluminescence with the BioRad^®^ ChemiDoc MP Imaging System to determine the relative optical densities. Pre-stained protein markers were used for molecular weight determinations. The intensity of the bands present in each lane was analyzed using BioRad^®^ Image Lab software (Bio-Rad Laboratories, Richmond, CA, USA) normalizing all measurements to the amount of total protein loaded in each well (thanks to the Stain Free technology of the Precast acrylamide Gels).

### 4.5. Enzyme-Linked Immunosorbent Assays

The levels of NLRP3 (#ER0800, FineTest, Wuhan Fine Biotech Co, Wuhan, Hubei, China) CASP1 and IL-18 (#MBS1600620 and #MBS8801271, Mybiosource, Vancouver, British Columbia, CA, USA) were determined in liver tissues using specific commercial enzyme-linked immunosorbent assays (ELISA) kits according to the manufacturer’s instructions.

### 4.6. Histological Staining

Liver tissues were fixed in a 10% formalin buffer solution for 24 h and samples were processed for embedding in paraffin. Serial sections (5 µm) were prepared using a rotary microtome Leica RM2125 RTS (Leica Biosystems, Wetzlar, Germany) for hematoxylin and eosin staining (H&E). The sections were stained with 0.1% hematoxylin (Ciba, Basel, Switzerland) for 5 min. Then slides were washed with tap water for 15 min and a quick wash with hydrochloric alcohol (0.5% HCl in absolute ethanol) to remove excess staining on the sample (differentiation). The acid was neutralized by immersing the sections in tap water for 5 min and a final wash with distilled water. They were immersed in 0.1% eosin (Ciba, Basel, Switzerland) for 5 min. After washing with distilled water, tissue sections were dehydrated using ascending ethanol passages and finished in xylol for 30 s. Images were captured with a Leica Microscope (Leica Biosystems, Wetzlar, Germany).

### 4.7. Statistical Analysis

Results were presented as mean ± SD. Means from more than two experimental groups were compared by 1-way analysis of variance (ANOVA). To account for multiple comparisons, the Tukey-Kramer multiple comparison test after testing for normal distribution. All statistical analyses were carried out with Prism v8 (GraphPad Software Inc., San Diego, CA, USA). Statistical significance was set at *p* < 0.05 in all the statistical analyses.

## 5. Conclusions

One of the BPA analogues that is replacing its use in plastic products is BPF. In this study, lactating dams treated with LBPF showed an increase in iNOS and HO-1d, activation of NLRP3 components, and promoted the release of proinflammatory cytokines. Similar effects were found in the offspring after perinatal exposure. The study found that BPF exposure caused an increase in nitrosative stress markers and proinflammatory cytokines. The activation of NLRP3 inflammasome was significantly increased in the liver of lactating dams and PND6 offspring. These findings suggest that BPF exposure can cause liver inflammation and may contribute to the development of liver disease.

## Data Availability

The data that support the findings of this study are available from the corresponding author upon reasonable request.
